# Fundamental Movement Skills and Their Assessment in Primary Schools from the Perspective of Teachers

**DOI:** 10.1080/1091367X.2021.1874955

**Published:** 2021-05-11

**Authors:** Lucy Eddy, Liam J.B. Hill, Mark Mon-Williams, Nick Preston, Andy Daly-Smith, Gareth Medd, Daniel D. Bingham

**Affiliations:** a School of Psychology, University of Leeds, LeedsUK, UK; b Bradford Institute for Health Research, Bradford Royal Infirmary, Bradford, UK; c Centre for Applied Education Research, Wolfson Centre for Applied Health Research, West Yorkshire, UK; d National Centre for Optics, Vision and Eye Care, University of South-Eastern Norway, Kongsberg, Norway; e Academic Department of Rehabilitation Medicine, University of Leeds, Leeds, UK; f Faculty of Health Studies, University of Bradford, Bradford, UK; g Center for Physically Active Learning, Faculty of Education, Arts and Sports, Western Norway University of Applied Sciences, Sogndal, Norway; h Institute of Education, University College London, London, UK; i Beckfoot Mutli-Academy Trust, Bradford, UK

**Keywords:** Fundamental movement skills, assessment, school, behavior change

## Abstract

Evidence suggests that children struggle to acquire age-appropriate fundamental movement skills (FMS), despite their importance for facilitating physical activity. This has led to calls for routine school-based screening of children’s FMS. However, there is limited research exploring schools’ capacity to conduct such assessments. This study investigated what factors might affect the adoption and implementation of FMS assessments in primary schools. School staff (n = 853) completed an online questionnaire developed using the Capability, Opportunity, Motivation and Behavior (COM-B) model. A majority reported that knowledge of pupils’ FMS ability would be beneficial (65.3%), and 71.8% would assess FMS if support was provided. Barriers included: Capability – few possessed knowledge of FMS (15%); Opportunity – teachers reported 30–60 minutes as acceptable for assessing a class, a substantially shorter period than current assessments require; Motivation – 57.2% stated FMS assessments would increase workload stress. Solutions to these issues are discussed using the COM-B theoretical framework.

## Introduction

Fundamental Movement Skills (FMS) is a term used to describe a group of motor behaviors which include locomotor, object manipulation and stability skills – for example, running, throwing and balancing-on-one-leg respectively (Rudd et al., 2015). Despite a focus on the development of FMS in the Early Years curriculum in the United Kingdom (UK) (Department For Education, 2014), formal screening and/or objective assessment of FMS is not common practice in schools. For example, in a child’s first year of formal schooling in the UK, teachers only record a single judgment of whether they feel a child’s 'moving and handling skills' are ‘above’, ‘at’, or ‘below’ expectation as part of the Early Years Foundation Stage Profile (EYFSP). Notably, however, the EYFSP is not based on any standardized measurement of FMS.

Meanwhile, there are grounds to suggest that failing to acquire FMS at the appropriate age may increase the risk of a child experiencing long-term physical and mental health problems (Barnett et al., 2016). FMS deficits are hypothesized to be causal in poor health because they influence a child’s ability to participate in physical activity (Burns et al., 2020; Jarvis et al., 2018; Logan et al., 2015, 2018; Stodden et al., 2008), and low levels of physical activity in childhood are associated with many adverse physical and mental health problems (Ahn & Fedewa, 2011; Timmons et al., 2012). Suggestion of a direct impact on educational attainment provides another mediating pathway through which FMS may influence childhood development; a recent systematic review (Macdonald et al., 2018) found evidence generally in favor of positive associations existing between FMS and educational attainment in reading and mathematics. Studies have also linked low levels of motor ability with social and emotional problems including: being withdrawn in social settings, having a poor self-concept, higher psychological distress, and increased anxiety levels (Brown & Cairney, 2020; Li et al., 2019a; Rodriguez et al., 2019).

Studies have suggested that a large proportion of children are unable to perform age-appropriate FMS (Brian et al., 2018; Farmer et al., 2017; O’Brien et al., 2016) and therefore specific and sensitive screening of FMS proficiency in schools may be valuable in helping to identify children with FMS deficits, opening up the possibility of providing additional targeted support. It is known that early identification of motor skill problems is beneficial (Missiuna et al., 2003), thus primary schools (or their international equivalent) would be an ideal location for assessments to identify children struggling with FMS development. We define Primary schools as the formal school that children in the UK attend between the ages of five and eleven years old. Empowering schools to assess FMS proficiency is also in line with current calls within the UK for schools to be pro-active in increasing physical activity levels (Department for Digital Culture Media & Sport, 2015; Department For Education, 2019).

However, whilst the proposition of assessing FMS in primary schools has a superficial appeal (e.g., helping mitigate issues with current assessment routes), there is no guarantee that assessing FMS in schools would be effective or feasible. For example, there are many assessment tools which market themselves as measuring FMS in school-aged children, yet a recent systematic review has shown that they are not suitable for use in schools in their current form (Eddy et al., 2020). Research has also shown that there are a wide range of barriers to implementing new initiatives in a school setting (Daly-Smith et al., 2020). One way of overcoming some of these barriers is through systematic consultation with teachers on the feasibility of school-based assessments. Previous research has looked at teacher perceptions of specific assessment tools after they have been trialed (Lander et al., 2016) and one previous study used interviews with a small sample size to understand teachers’ more general opinions on school-based assessments of FMS (Van Rossum et al., 2019). However, to date, no research has utilized evidence-based theoretical behavioral science frameworks to understand teachers’ current skills, and schools’ capacity to implement and benefit from such assessments. This consideration is an essential first step in detailing the ‘lie of the land’ within schools, to intelligently inform the process of identifying, designing, adapting, and then trialing school-based FMS assessments.

Previous research has highlighted the importance of using behavior change models when embedding initiatives within schools (Daly-Smith et al., 2020). The Capability, Opportunity, Motivation and Behavior (COM-B) model of behavior change (Michie et al., 2011) is one theory that can be applied in this context. The COM-B model suggests that behaviors occur as a result of an individual’s capability, opportunity and motivation to perform them. Capability can be either psychological (e.g., knowledge) or physical (e.g., skills), opportunity can be social (e.g., societal influences) or physical (e.g., environmental resources), and motivation can be automatic (e.g., emotion) or reflective (e.g., intentions and goals). The COM-B model proposes that behavior change at an individual, organizational, and/or population level has a greater likelihood of occurring when these three facilitatory components are enhanced. Thus, to understand how to encourage universal screening in schools (the behavior of interest), we first need to understand the current capability (e.g., teachers’ ability to demonstrate FMS), opportunity (e.g., time within the curriculum to assess these skills) and motivation (e.g., belief about the benefits of FMS assessments) of teachers to host such assessments. The COM-B model is underpinned by a complex behavior structure, the Theoretical Domains Framework (TDF), which consolidated 33 behavior change theories into 14 key factors which can influence behavior. The TDF factors all tie in with the three COM-B components (Cane et al., 2012). Due to this synthesis, utilizing the COM-B model is beneficial because it allows understanding of a wide range of multifaceted factors influencing behavior(s) through using one model of behavior change, rather than applying multiple theories or being more selective of theories. Additionally, Michie et al. (2011) have since matched behavior change techniques (Behavior Change Wheel) to the COM-B model and the TDF, which proposes solutions to increase the likelihood of a behavior (e.g., implementing FMS assessments in schools) occurring, such as training and incentivizing initiatives.

The COM-B model (Michie et al., 2011) and the TDF (Cane et al., 2012) in combination with the Behavior Change Wheel, provide a sound theoretical foundation which can be applied to identifying what factors may affect the adoption and implementation of universal screening in schools in a more comprehensive way. It also underpins these investigations with theoretical evidence and advises on behavior change techniques that can be used to overcome barriers that are subsequently identified. The current study therefore used these models and frameworks to collect data from teachers and other educators, in order to investigate what factors might influence teachers’ capability, opportunity and motivation to implement assessments of FMS in schools, to help make inferences about barriers and facilitators of universal screening in these settings.

## Materials and methods

### Participants and procedure

Teachers or staff who worked in a Primary school in a role which directly supports the education of pupils (e.g., head teachers, teaching assistants) were invited to take part in an online questionnaire. This population was selected due to a lack of P.E. specialization within primary schools in the UK (Ofsted, 2013), which means it is likely that the responsibility could be placed upon any member of teaching staff if schools were required to universally screen FMS. The questionnaire, which had 29 items, was hosted by Qualtrics (www.qualtrics.com/uk/), and was advertised on social media (e.g., teacher groups and forums on Facebook and Twitter) as well as through links with local schools. Participants were entered into a prize draw that gave them a chance to win one of three £20 “Amazon.co.uk” vouchers as an incentive for taking part in this study. The questionnaire took participants approximately ten minutes to complete, and was available online between February and July 2019. Ethical approval for this study was granted by the University of Leeds School of Psychology Ethics Committee (reference: PSC-591).

### Measure – online questionnaire

Demographic information was collected about participants’ gender, age, highest qualification, age groups taught, job role, years of teaching experience, type of school, country, and whether they had received training on FMS prior to completing the questionnaire. Questions were based on previous research which explored the feasibility of FMS assessments for use in schools (Klingberg et al., 2018a) and were mapped alongside all six sub-elements within the COM-B model and categorized in relation to the Theoretical Domains Framework (TDF) (Cane et al., 2012). There was extensive discussion amongst authors on the wording of the questions to ensure that they were both easily comprehensible and theoretically driven. Categorizations for the COM-B model and the TDF were discussed and agreed upon amongst authors. Disagreements amongst authors were resolved through consultation with a behavior change researcher who was not involved with the design of the questionnaire. Multiple choice, scale and rank questions were used to explore primary school teachers’ opinion of their capability (e.g., ability to demonstrate FMS to pupils), opportunity (e.g., senior leadership team support for such initiatives) and motivation (e.g., how beneficial they believe knowledge of their pupils’ FMS levels would be for their teaching) to assess FMS. For a full breakdown of questions included in the questionnaire, and the aspects of the COM-B model and TDF framework they align with, see table 1.

**Table 1.** Questionnaire items in relation to the Capability, Opportunity, Motivation and Behavior (COM-B) model of behavior change (Michie et al., 2011) and the Theoretical Domains Framework (TDF; Cane et al., 2012)

			**Construct Measured**	
**Variable**	**Questionnaire item**	**Reponses**	**Component of COM-B model**	**Component of TDF**
Perceived knowledge	How knowledgeable do you think you are about motor skills that are defined as ‘Fundamental Movement Skills’?	1)Not knowledgeable at all, 2), 3), 4), 5)Extremely knowledgeable	Capability (psychological)	Knowledge
Actual knowledge	Which of the following motor skill do you think comprise ‘Fundamental Movement Skills’?	Running, Handwriting, Hopping, Jumping, Using cutlery, Balancing, Dressing oneself, Throwing, Catching, Kicking, Brushing teeth, Riding a bike, Swimming	Capability (psychological)	Knowledge
Knowledge of relationship between FMS and outcomes	On a scale of 1–5, to what extent do you think the development of fundamental movement skills has an impact upon: Academic attainment? Participation in PA? Mental Health? Physical Health? Social Relationships?	1)No impact at all, 2) … .3) … .4) … .5)Large impact	Capability (psychological)	Knowledge
Confidence Demonstrating	On a scale of 1–5, how confident are you that you could demonstrate the following activities: Running between two markers for 15 seconds? Throwing beanbags into a target box two meters away? Hopping between two markers one meter apart? Holding a balance (e.g., standing on one leg) whilst passing a beanbag around your body?	1)Not confident at all, 2) … 3) … 4) … 5)Extremely Confident	Capability (physical)	Physical Skills
Confidence Assessing	On a scale of 1–5, how confident are you that yourself and one other member of staff could assess five children simultaneously in the following activities: Running between two markers for 15 seconds? Throwing beanbags into a target box two meters away? Hopping between two markers one meter apart? Holding a balance (e.g., standing on one leg) whilst passing a beanbag around your body?	1)Not confident at all, 2) … 3) … 4) … 5)Extremely Confident	Capability (physical)	Physical skills
Assessment of FMS in school	Do you/your school currently assess fundamental movement skill proficiency?	Yes, No, Unsure	Opportunity (physical)	Environmental context and resources
Support from senior leadership	Do you think the senior leadership team at your school would be supportive if you wanted to assess fundamental movement skill proficiency in your class?	Definitely yes, Probably yes, Probably not, Definitely not	Opportunity (social)	Social influences
Access to additional support staff resource	Would you be able to access support from another member of staff (e.g., teaching assistant) to help you deliver an assessment of fundamental movement skills to a whole class?	Definitely yes, Probably yes, Probably not, Definitely not	Opportunity (physical)	Environmental context and resources
Access to equipment	Does your school have the following equipment: 25 beanbags? Chalk? A sports hall larger than 5 m x 5 m? Outdoor space larger than 5 m x 5 m? Stop watch? Tape measure or meter ruler?	Yes, No, Unsure	Opportunity (physical)	Environmental context and resources
Acceptable assessment time	Over the course of a single school week, once per academic year, how long do you think is acceptable to spend assessing the fundamental movement skills of: one child a whole class?	Per Child: < 10 minutes, 10–30 minutes, 30–60 minutes, 60–90 minutes, Up to 2 hours, 2–3 hours, 3 hours + Per class: < 10 minutes, 10–30 minutes, 30–60 minutes, 60–90 minutes, Up to 2 hours, 2–3 hours, 3 hours +	Opportunity (physical)	Environmental context and resources
2 hour start of school year assessment	Do you think you have would be able to make time in the curriculum to spend two hours at the start of the school year evaluating your class’ fundamental movement skills?	Definitely yes, Probably yes, Probably not, Definitely not	Opportunity (physical)	Environmental context and resources
Time in school day most suitable to assess FMS	What time of the day would you be most likely be able to find time to assess fundamental movement skills?	Physical Education (P.E.) lessons, Core lessons (Math, English and Science), Other lessons (e.g., Languages and Art), After school, Before school	Opportunity (physical)	Environmental context and resources
Perceptions of ability to identify children who need support through FMS assessment in schools	Do you think a school based assessment of fundamental movement skills has the ability to identify children who need additional support?	Yes, No, Maybe	Motivation (reflective)	Optimism
Perceived benefit of knowledge of pupils’ FMS for teaching	On a scale of 1–5, how beneficial to your teaching would it be to have knowledge about your pupils’ fundamental movement skills?	1)Not beneficial at all, 2) … 3) … 4) … 5)Extremely beneficial	Motivation (reflective)	Beliefs about consequences
Workload stress	Do you think that assessing childhood fundamental movement skills in school would increase your workload stress?	Definitely yes, Probably yes, Probably not, Definitely not	Motivation (automatic)	Emotion
Likelihood of assessing FMS	On a scale of 1–5, if you had training and support available, how likely would you be to assess the fundamental movement skills of the children in your class?	1)Not likely at all, 2) … .3) … 4) … .5)Extremely likely	Motivation (reflective)	Intentions & beliefs about capabilities
Peer influence	How likely would your decision regarding whether to assess the fundamental movement skills be influenced by the opinions of other teachers in your school?	1)Not likely at all, 2) … .3) … 4) … .5)Extremely likely	Motivation (reflective)	Professional/social role and identity

*NB*: For confidence demonstrating and assessing FMS authors decided to include at least one of each type of FMS. The four example skills were included as they are regularly assessed by popular assessment tools, including the MABC (Hendersen, Sugden & Barnett, 2007) and the BOT (Bruininks & Bruininks, 2005). Two locomotor skills were included as both have very different difficulty levels. Hopping is a more advanced locomotor skill which requires greater strength, and better vestibular and motor control. It is therefore likely to be more difficult for adults to demonstrate, particularly less fit adults, those that are overweight or those with lower limb injuries or medical conditions such as osteoarthritis.

### Data analysis

Patterns observed in the descriptive statistics were explored and multinomial logistic regression was used to investigate whether there were any relationships between demographic factors and responses to each question. Gender, age, highest qualification, years of teaching experience, job role, school type, and whether respondents had received training on FMS were all included in the regression models. For age, categories 5 and 6 (56–65 years and 66+ years) were combined with age category 4 (46–55 years) as only seventeen participants were over the age of 55 years. The country in which respondents taught was not included in the regression model as the number of responses from outside of the UK was too low to test differences of opinion and make meaningful conclusions. Age groups taught were not included in the analysis as respondents often selected more than one age group. The significance level was set at *p* ≤ .001 to account for multiple testing. All analyses were conducted using Statistical Package for the Social Sciences (SPSS) version 24.

## Results

The questionnaire was online for 133 days. A total of 1074 people opened and began filling in the questionnaire; 221 people did not complete the questionnaire and their responses were therefore excluded.

### Participants

A total of 853 primary school staff fully completed the survey and had their data analyzed. Participant demographics are given in table 2. Participants reported working across 32 different countries, including the UK (*n*= 746, 87.7%), India (*n*= 10, 1.2%), the USA (*n = *7, 0.8%) as well as Australia, Germany, Ireland and Malta which all had five responses (0.6%). The remaining responses spanned six continents: Africa (7 responses from 5 countries), Asia (20 responses from 15 countries), Europe (9 responses from 7 countries), North America (3 responses from 2 countries), Oceania (3 responses from 2 countries) and South America (1 response from Mexico). The mean time spent in a teaching role was 8.57 years (*SD* = 7.71, range = 2 months – 45 years 3 months). The most common responses when job role was selected as ‘other’ were: deputy headteacher (*n*= 19, 2.2%), trainee teacher (*n*= 8, 0.9%), head of year/phase (*n*= 8, 0.9%), higher level teaching assistant (HLTA; *n*= 7, 0.8%). When ‘other’ was selected for type of school, the most common responses were: special educational needs schools (*n*= 9) and faith schools (*n*= 5). Only 128 primary school staff (15.1%) claimed to have received training on FMS, ranging from lectures within degrees to programmes used within schools to knowledge disseminated from Physical Education (P.E.) leads in their schools.

**Table 2.** The demographic characteristics of the school workers that complete the online questionnaire

**Demographic Variable**	***n***	**%**
**Gender**		
Male	54	6.4
Female	788	92.9
Prefer not to say	6	0.7
**Age**		
18–25	170	20
26–35	345	40.6
36–45	203	23.9
46–55	113	13.3
56–65	17	2
66+	1	0.1
**Highest Qualification**		
General Certificate of Secondary Education	7	0.8
Advanced Subsidiary Level	2	0.2
Advanced Level	26	3.1
Undergraduate degree	280	32.9
Masters Degree	89	10.4
Professional Degree (e.g., PGCE)	441	52.1
Doctoral Degree	2	0.2
**Job Role**		
Teacher	701	82.3
Teacher Assistant	37	4.3
Headteacher	21	2.5
Special Educational Needs Coordinator	58	6.8
Other	83	9.7
**Age Groups of Children Taught**		
4–5 years	204	23.9
5–6 years	221	25.5
6–7 years	217	25.4
7–8 years	262	30.8
8–9 years	269	31.6
9–10 years	224	26.3
10–11 years	216	25.4
**Type of School Taught In**		
State	543	64.1
Private	66	7.8
Academy	212	25
Other	26	3.1
**Training on FMS**		
Yes	128	15.1
No	719	84.4

### Capability

Frequencies for responses to capability questions are reported in full in table 3.

**Table 3.** Responses to questions designed to measure the capability of teachers to assess fundamental movement skills in a school setting

**Variable**	***n***	**%**
**Perceived knowledge of FMS**		
1 (Not knowledgeable at all)	225	26.6
2	322	38
3	254	30
4	43	5.1
5 (Extremely knowledgeable)	3	0.4
**Knowledge of FMS**		
Running	615	72.2
Handwriting	317	37.2
Hopping	553	64.9
Jumping	626	73.5
Using cutlery	351	41.2
Balancing	736	86.4
Dressing oneself	371	43.5
Throwing	554	65
Catching	544	63.8
Kicking	489	57.4
Brushing teeth	290	34
Riding a bike	219	25.7
Swimming	214	25.1
All correct	356	48.1
All correct no incorrect	128	15
All answers on the list	111	13
All incorrect	118	13.8
All incorrect no correct	1	0.1
**Knowledge of relationship between FMS and outcomes**		
*Academic Attainment*		
1 (No impact at all)	3	0.4
2	34	4
3	223	26.3
4	350	41.1
5 (Large impact)	239	28.1
*Physical Activity*		
1 (No impact at all)	2	0.2
2	11	1.3
3	53	6.2
4	203	23.8
5 (Large impact)	579	68.3
*Mental Health*		
1 (No impact at all)	2	0.2
2	31	3.6
3	141	16.5
4	371	43.5
5 (Large impact)	301	35.6
*Physical Health*		
1 (No impact at all)	2	0.2
2	23	2.7
3	79	9.3
4	281	33
5 (Large impact)	462	54.2
*Social Relationships*		
1 (No impact at all)	8	0.9
2	57	6.7
3	220	25.8
4	385	45.2
5 (Large impact)	177	20.8
**Confidence Demonstrating**		
*Running between two markers*		
1 (not confident at all)	1	0.1
2	12	1.4
3	62	7.3
4	152	17.8
5 (extremely confident)	621	72.9
*Throwing beanbags to a target*		
1 (not confident at all)	2	0.2
2	12	1.4
3	121	14.2
4	242	28.4
5 (extremely confident)	472	55.4
*Hopping between two markers*		
1 (not confident at all)	5	0.6
2	21	2.5
3	94	11
4	194	22.8
5 (extremely confident)	531	62.3
*Holding a balance whilst passing a beanbag*		
1 (not confident at all)	4	0.5
2	37	4.3
3	132	15.5
4	227	26.6
5 (extremely confident)	446	52.3
**Confidence assessing**		
*Running between two markers*		
1 (not confident at all)	1	0.1
2	28	3.3
3	176	20.7
4	278	32.6
5 (extremely confident)	363	42.6
*Throwing beanbags to a target*		
1 (not confident at all)	1	0.1
2	25	2.9
3	133	15.6
4	300	35.2
5 (extremely confident)	388	45.5

#### Perceived knowledge

Perceived knowledge about FMS was relatively low, only 5.5% claimed to be either ‘very’ (*n*= 44, 5.1%) or ‘extremely’ (*n*= 4, 0.4%) knowledgeable. A large proportion (68%) did believe they had ‘some’ working knowledge of FMS. A multinomial regression showed that the final model was a better fit with demographic factors included than the intercept only model (*χ*2 (80) = 233.7, *p*< .001). Only previous teacher training in FMS predicted a positive response to perceived knowledge (*χ*2(4) = 145.83, *p*< .001) at the accepted significance level (see table 4). Respondents who had received training on FMS were more likely to think that they had greater knowledge of FMS than those who had not received training. Using the response ‘not knowledgeable at all’ as the reference category, teaching staff that had received training were 29 times more likely to select ‘moderately knowledgeable’ (*OR* = 29.26, *CI* = 8.99–95.28), 117 times more likely to believe they were ‘very knowledgeable’ (*OR* = 117.30, *CI* = 31.08–442.70), and 182 times more likely to think they were ‘extremely knowledgeable’ (*OR* = 182.43, *CI* = 9.02–3691.61).

**Table 4.** Likelihood Ratio Tests for teachers’ perceived knowledge of fundamental movement skills

**Effect**	***χ2***	***df***	***p***
Intercept	.00	0	
Teaching Experience (years)	.134	4	.99
Type of School	15.41	12	.22
Training	145.83	4	<.001
Sex	18.10	8	.02
Highest Qualification	21.45	24	.61
Age Group	6.45	12	.89
Job Role	13.07	16	.67

NB: Accepted level of significance was p ≤ .001

#### Actual knowledge

When asked to select from a list of motor skills only those that are classified as FMS, 355 (42%) of the respondents selected all the correct answers (running, jumping, hopping, throwing, kicking, catching and balancing). However, 227 of this subsample (63.9%) also selected at least one incorrect answer. The most commonly selected incorrect answers were ‘activities of daily living’ including dressing oneself (43.5%), using cutlery (41.2%) and brushing one’s teeth (34%). None of the demographic factors were predictors for knowledge of what skills comprise FMS (*χ*2 (80) = 170.47, *p*= .04).

#### Knowledge of relationship between FMS and outcomes

There was a fairly good understanding of the relationships between FMS and childhood development, with 69.2% of respondents (*n*= 589) agreeing that FMS had a moderate or large impact on academic attainment, 66% (*n*= 562) on social relationships and 79.1% (*n*= 671) on mental health. Teaching staff perceptions of the impact of FMS on physical activity and physical health were greater still at 92% (*n = *782) and 87% (*n =* 743) respectively. Multinomial regressions found that the final model with demographics included was not a better predictor of responses to the impact of FMS on physical activity (*χ*2 (80) = 72.33, *p*= .87), mental health (*χ*2 (80) = 78.55, *p*= .53) or physical health (*χ*2 (80) = 68.43, *p*= .82). Analyses found that the final model was a better predictor of responses to the importance of FMS for academic attainment (*χ*2 (80) = 131.22, *p*< .001), and social relationships (*χ*2 (80) = 164.29, *p*< .001), however, none of the demographic variables alone significantly predicted responses for academic attainment (see table 5). For social relationships, age group predicted responses (see table 6), in which age groups one (18–25 years) were seven times more likely to state that FMS had a ‘moderate impact’ on social relationships than a ‘very large impact’ when compared to all other age groups (*OR* = 7.07, *CI *= 2.67–18.75).

**Table 5.** Likelihood Ratio Tests for the perceived impact of fundamental movement skills on academic attainment

**Effect**	***χ2***	***df***	***p***
Intercept	.00	0	
Teaching Experience (years)	8.12	4	.09
Type of School	15.90	12	.20
Training	3.44	4	.49
Sex	13.87	8	.09
Highest Qualification	20.44	24	.67
Age Group	17.71	12	.13
Job Role	15.05	16	.52

NB: Accepted level of significance was p ≤ .001

**Table 6.** Likelihood ratio tests for the perceived impact of fundamental movement skills on social relationships

**Effect**	***χ2***	***df***	***p***
Intercept	.00	0	
Teaching Experience (years)	6.52	4	.16
Type of School	24.44	12	.02
Training	2.31	4	.68
Sex	8.31	8	.40
Highest Qualification	27.84	24	.27
Age Group	31.99	12	.001
Job Role	31.33	16	.01

NB: Accepted level of significance was p ≤ .001

#### Confidence demonstrating

When asked to rate their ability to demonstrate FMS on a scale between one and five (with one indicating ‘not confident at all’ and five indicating ‘extremely confident’), 92.1% (n *= *786) were confident (selecting responses four or five) that they could run between two markers for 15 seconds. Confidence was also high for throwing into a target box (*n*= 717, 84.1%), hopping between two markers (*n*= 732, 85.8%), and holding balance (*n =* 679, 79.6%). Demographic factors did not play a significant role in responses to confidence demonstrating ‘running’ (*χ*2(80) = 81.54, *p*= .43), ‘throwing’ (*χ*2(80) = 80.02, *p*= .49), ‘hopping’ (*χ*2(80) = 79.1, *p*= .51) or ‘balance’ (*χ*2(80) = 36.44, *p*= 1.00).

#### Confidence assessing

When asked about confidence in assessing small-groups (of five) children simultaneously for the activities described above, confidence rates remained positive, with 75.8% (*n*= 647) responding with four or five on the scale for ‘running’, 81.2% (*n*= 693) for ‘throwing’, 77.5% (*n*= 661) for ‘hopping’ and 75.3% (*n*= 642) for ‘balancing’. Demographic factors did not play a significant role in responses to confidence assessing five children at once for ‘running’ (*χ*2(80) = 49.49, *p*= .43), ‘throwing’ (*χ*2(80) = 91.55, *p*= .18), ‘hopping’ (*χ*2(80) = 83.58, *p*= .37) or ‘balance’ (*χ*2(80) = 114.14, *p*= .007).

### Opportunity

Frequencies for responses to opportunity questions are reported in full in table 7.

**Table 7.** Responses to questions designed to understand the opportunity for teachers to assess fundamental movement skills in a school setting

**Variable**	***n***	**%**
**Assessment of FMS in school**		
Yes	128	15
No	403	47.3
Unsure	317	37.2
**Support from senior leadership**		
Definitely yes	212	24.9
Probably yes	524	61.5
Probably not	109	12.8
Definitely not	3	0.4
**Access to additional support staff resource**		
Definitely yes	276	32.4
Probably yes	387	45.4
Probably not	149	17.5
Definitely not	36	4.2
**Access to equipment**		
*25 beanbags*		
Yes	696	81.7
No	77	9
Unsure	75	08.8
*Chalk*		
Yes	774	90.8
No	35	4.1
Unsure	38	4.5
*Sports hall larger than 5 × 5 meters*		
Yes	741	87
No	69	8.1
Unsure	37	4.3
*Outdoor space larger than 5 × 5 meters*		
Yes	832	97.9
No	11	1.3
Unsure	5	0.6
*Stopwatch*		
Yes	789	92.3
No	25	2.9
Unsure	37	4.3
**Acceptable assessment time**		
*Per child*		
<10 mins	393	46.1
10–30 mins	327	38.4
30–60 mins	73	8.6
60–90 mins	13	1.5
Up to 2 hours	8	0.9
2–3 hours	3	0.4
3 hours+	2	0.2
*Whole class*		
<10 mins	5	0.6
10–30 mins	80	9.4
30–60 mins	205	24.1
60–90 mins	166	19.5
Up to 2 hours	132	15.5
2–3 hours	113	13.3
3 hours+	132	15.5
**Twohour start of schoolyear assessment**		
Definitely yes	194	22.8
Probably yes	478	56.1
Probably not	157	18.4
Definitely not	18	2.1
**Time in schoolday most suitable to assess FMS**		
PE lessons	730	85.7
Core lessons	22	2.6
Other lessons	17	2
After school	13	1.5
Before school	20	2.3

#### Assessment of FMS in schools

When teaching staff were asked whether they personally, or their school, currently assess their pupils’ FMS, 128 people (15%) in the sample responded with ‘yes’, 398 (47.6%) stated they did not, and 319 (37.4%) were unsure. A multinomial logistic regression found that a model with all demographic factors included was a better predictor of responses than a model without these factors (*χ*2(40) = 129.75, *p*< .001). Previous FMS training was the only factor to predict responses to this question (*χ*2(2) = 36.57, *p*< .001) (see table 8). Teaching staff that had previously completed training on FMS were four times more likely to say that they, or their school, currently assess the FMS of their pupils (*OR *= 4.19, *CI* = 2.54–6.91).

**Table 8.** Likelihood Ratio Tests for Whether Schools Currently Assess fundamental movement skills

**Effect**	***χ2***	***df***	***p***
Intercept	.00	0	
Teaching Experience (years)	3.61	2	.17
Type of School	4.63	6	.59
Training	36.57	2	<.001
Sex	3.83	4	.43
Highest Qualification	21.00	12	.05
Age Group	9.82	6	.13
Job Role	19.52	8	.01

NB: Accepted level of significance was *p*≤ .001

#### Support from senior leadership

A large proportion of teaching staff (*n*= 736, 86.4%) believed that senior leadership teams (SLT) in their school would ‘definitely’ or ‘probably’ be supportive if they decided they would like to assess the FMS proficiency of their pupils. None of the demographic variables were predictors of teacher perceptions of SLT support (*χ*2(80) = 97.72, *p*= .002).

#### Access to additional support staff resource

The majority of respondents believed they would ‘definitely’ (*n* = 277, 32.5%), or ‘probably’ (*n*= 389, 45.6%) be able to enlist another member of staff to help them to assess FMS proficiency in school. Only 4.2% of the sample (*n*= 36) claimed that this would ‘definitely not’ be possible. Analyses revealed that the intercept only model was not improved by including demographic factors for this question (*χ*2(60) = 79.97, *p*= .04).

#### Access to equipment

When asked whether schools had access to basic equipment that would enable the testing of FMS, the majority of staff said their schools had ‘25 beanbags’ (*n*= 696, 81.7%), ‘chalk’ (*n*= 774, 90.8%), a ‘sports hall larger than five meters squared’ (*n*= 741, 87%), an ‘outdoor space larger than five meters squared’ (*n*= 832, 97.7%), a ‘stopwatch’ (*n*= 786, 92.3%) and a ‘tape measure or meter ruler’ (*n*= 827, 97.1%). None of the demographics was predictive of teacher responses to access to equipment in schools: ‘25 beanbags’ (*χ*2(40) = 54.93, *p*= .06), ‘chalk’ (*χ*2(40) = 53.99, *p*= .07), a ‘large enough sports hall’ (*χ*2(40) = 52.67, *p*= .09), ‘suitable outdoor space’ (*χ*2(40) = 57.76, *p*= .03), a ‘stopwatch’ (*χ*2(40) = 34.97, *p*= .70), and a ‘tape measure’ (*χ*2(40) = 30.96, *p*= .85).

#### Acceptable assessment time

School staff were also asked how long would be acceptable to spend assessing the FMS of one child and a whole class at the start of the academic year, with the most common responses being ‘less than ten minutes’ and ‘30–60 minutes’, respectively. Demographic factors were not predictors for acceptable time to assess FMS per child (*χ*2(120) = 59.38, *p*= 1.00) or for a whole class (*χ*2(120) = 125.32, *p*= .35).

#### Two hour start of year assessment

The majority of teaching staff said that they would be able to devote two hours at the start of the school year to assessing FMS, selecting either ‘definitely yes’ (*n*= 194, 22.8%) or ‘probably yes’ (*n*= 478, 56.1%). Only 18 participants (2.1%) stated that this would ‘definitely not’ be possible. A multinomial logistic regression found that the final model significantly predicted responses better (*χ*2(60) = 102.85, *p*< .001). Whether or not teaching staff had received training on FMS previously was the only demographic factor that had a significant impact upon responses (*χ*2(3) = 20.01, *p*< .001) to this question (see table 9). Further exploration showed that teaching staff that had received training were 62% less likely to say ‘probably yes’ than ‘definitely yes’ (*OR* = .38, *CI* = .24 – .60).

**Table 9.** Likelihood Ratio Tests for whether teaching staff would be able to spend 2 hours at the start of the school year assessing the fundamental movement skills of their pupils

**Effect**	***χ2***	***df***	***p***
Intercept	.00	0	
Teaching Experience (years)	5.76	3	.12
Type of School	20.22	9	.02
Training	20.01	3	<.001
Sex	8.80	6	.19
Highest Qualification	17.51	18	.49
Age Group	9.79	9	.37
Job Role	8.27	12	.76

NB: Accepted level of significance was p ≤ .001

#### Time in school day most suitable to assess FMS

When asked to rank when they would most likely be able to find time to assess FMS in schools, the most popular response was ‘during P.E. lessons’ (91%). The least feasible time to assess these skills was ‘before school’, with 41.5% of the sample ranking this last. Demographic factors did not play a significant role in responses to this question (*χ*2(80) = 76.21, *p* = .60).

### Motivation

Frequencies for responses to motivation questions are reported in full in table 10.

**Table 10.** Responses to questions designed to measure the motivation of teachers to assess fundamental movement skills in a school setting

**Variable**	***n***	**%**
**Perceptions of ability to identify children who need support through FMS assessment in schools**		
Yes	618	72.5
No	14	1.6
Maybe	216	25.4
**Perceived benefit of knowledge of pupils’ FMS for teaching**		
1 (not beneficial at all)	2	0.2
2	42	4.9
3	251	29.5
4	322	37.8
5 (extremely beneficial)	229	26.9
**Workload stress**		
Definitely yes	94	11
Probably yes	394	46.2
Probably not	330	38.7
Definitely not	30	3.5
**Likelihood of assessing FMS**		
1 (not likely at all)	3	0.4
2	45	5.3
3	190	22.3
4	322	37.8
5 (extremely likely)	285	33.5
**Peer influence**		
1 (not likely at all)	44	5.2
2	84	9.9
3	226	26.5
4	380	44.6
5 (extremely likely)	114	13.4

#### Perception of ability to identify children who need support through FMS assessment in schools

The majority of school staff believed that a school-based assessment would be able to identify children who need extra support (72.9% yes, 25.5% maybe), with only 1.4% of the sample claiming they did not think this would be the case. Demographic factors did not play a significant role in responses to confidence in identifying children who need extra support (*χ*2(40) = 67.92, *p*= .004).

#### Perceived benefit of knowledge of pupils’ FMS for teaching

When asked to rate on a scale from one (not beneficial at all) to five (extremely beneficial) whether their teaching would benefit if they were aware of their pupils’ FMS ability, only 5.2% of school staff responded with either one or two. The majority of respondents selected either three (29.7%), four (38.1%) or five (27.2%). Demographic factors were found to significantly predict responses (*χ*2(80) = 143.34, *p*< .001). Both training (*χ*2(4) = 23.84, *p*< .001) and job role (*χ*2(16) = 55.97, *p*< .001) were predictive of the way respondents answered (see table 11).

**Table 11.** Likelihood Ratio Tests for perceived benefit of knowledge of pupils’ fundamental movement skills for teaching

Effect	χ2	df	p
Intercept	.00	0	
Teaching Experience (years)	6.54	4	.16
Type of School	21.41	12	.05
Training	23.84	4	<.001
Sex	8.28	8	.41
Highest Qualification	25.87	24	.36
Age Group	16.04	12	.19
Job Role	55.97	16	<.001

NB: Accepted level of significance was p ≤ .001

#### Workload stress

When asked whether assessing FMS in schools would increase workload stress, over half of the respondents selected ‘definitely yes’ (*n*= 94, 11%) or ‘probably yes’ (*n*= 394, 46.2%). Only 30 participants selected ‘definitely not’ (3.5%). Demographic factors did not have a significant effect on the regression model (*χ*2(60) = 87.21, *p* = .01).

#### Peer influence

When asked whether their decision to assess FMS would be influenced by the opinion of other staff in their school, over half of the respondents selected either ‘extremely likely’ (*n*= 114, 13.4%) or ‘somewhat likely’ (*n*= 380, 44.6%), and only 15.1% of participants selected that it would be ‘not likely at all’ (5.2%, *n*= 44) or ‘somewhat unlikely’ (9.9%, *n*= 84) to influence them. Demographic factors did not play a significant role in how participants responded to this question (*χ*2(80) = 109.59, *p* = .02).

#### Likelihood of assessing FMS

When asked on a scale of one (not likely at all) to five (extremely likely) how likely they would be to assess the FMS proficiency of their pupils if they had appropriate training and support available, the response was largely positive, with 71.8% of the sample choosing four or five, and thus being likely to implement such an initiative. Only 5.7% of the sample (*n*= 47) selected one or two, indicating they would be unlikely to assess their pupils’ FMS. Demographic factors did not have a significant effect on the regression model (*χ*2(80) = 97.50, *p* = .09).

## Discussion

For the first time, a behavior change framework was utilized to understand what factors may influence teachers’ capability, opportunity and motivation to implement assessments of FMS in schools, helping to clarify potential factors which may bias the adoption and implementation of universal screening in these settings. Thelarge number of teaching staff sampled offers a unique insight into the challenges that schools might face when attempting to introduce an assessment of FMS into their curriculum. Encouragingly, the responses demonstrate a large appetite for school-based assessments, with many believing that such initiatives could help to identify children who need extra support, whilst also aiding teachers. Despite this, only 15% of respondents were confident that such assessments already take place in their school. Using the COM-B model (Michie et al., 2011) alongside these insights enables behavior change techniques to be paired with barriers to identify practical solutions for a school setting.

Results are in line with a previous, much smaller, study that showed knowledge is a barrier to school-based assessments of FMS (Van Rossum et al., 2019). Approximately a quarter of teachers surveyed here indicated low or no perceived knowledge of FMS. This apparent gap in teachers’ toolboxes was also highlighted by low levels of accuracy in discriminating movements defined as FMS. This finding is, perhaps, unsurprising as 85% of the sample do not recall having training on FMS. The lack of FMS in teacher training courses is particularly alarming, due to the wide-ranging impacts this group of motor skills has on childhood development (Ahn & Fedewa, 2011; Brown & Cairney, 2020; Burns et al., 2020; Li et al., 2019a; Stodden et al., 2008; De Waal, 2019). Additionally, the results of this questionnaire found that teachers who had previous training on FMS were more likely to work in schools where FMS assessments are being undertaken, and were also more likely to think that there would be sufficient time for a start of year assessment and that results of such assessments would aid teaching practices. These responses, collectively, highlight that school-based FMS assessment tools will need to incorporate a teacher training session that educates staff on the rationale for testing FMS, if school-based assessments are to become a reality. Further behavior change techniques that can be applied to ameliorate knowledge barriers include restructuring the social and physical environment (Michie et al., 2011). One way social barriers could be addressed is through ensuring that staff training is conducted specifically in a group setting. This would help create a culture of understanding about FMS and the role they play within a school environment. This training may play a particularly important role for schools which do not have a P.E. lead, and would require teachers without this specialism to conduct the assessments. Class teachers will need to be aware of the implications of poor FMS for other aspects of their school life so that they can be supported appropriately in a classroom setting, even if they are not directly assessing these skills. Research has shown that having senior leadership support new initiatives is beneficial to teachers’ development (Taylor et al., 2011), so ensuring members of the senior leadership team (SLT) are present during training may also be crucial. Additionally, in order to ensure that knowledge is retained, physical prompts should be provided to the school following training sessions. For example, placing this key contextual information at the front of a manual that explains the assessment tool. These methods have previously been found to be highly effective for teacher-led FMS interventions, in which teachers received both face-to-face training and resources to utilize afterward (Brian et al., 2017).

Understanding barriers to school-based assessment of FMS must go beyond addressing shortcomings in knowledge though. A further barrier that was highlighted was the duration of assessments. Uniquely, the results revealed challenges with using pre-existing FMS assessments within the school setting. Teachers identified 30 to 60 minutes as a maximum time to assess a whole class, yet, current assessment tools require such durations per individual child (Klingberg et al., 2018b). This highlights a gulf between current approaches and needs of schools, who have limited time and significant pressures in other areas of their provision. Additionally, while the majority of schools possessed basic equipment that could be used to assess FMS (e.g., beanbags), it is important to note that current assessment tools are burdensome on already pressured school budgets (Turner et al., 2017), often costing £500-1000 to purchase specific copyrighted resources. As these factors are unlikely to change within schools, the physical requirements of FMS assessment tools will need to be modified (Michie et al., 2011). In order for school-based FMS initiatives to become a reality, it will therefore be important that measures utilize equipment which is readily available in schools (Klingberg et al., 2018b), and ensure that a whole class can be assessed within the time and space constraints of a P.E. lesson.

The importance of ensuring a supportive social environment in schools to enable the introduction of FMS assessments in schools was highlighted by the fact that over half of the sample perceived the opinion of other staff to be important to making the decision to assess FMS. Encouragingly, over three quarters of respondents believed that both immediate colleagues, such as teaching assistants, and senior school leaders would support FMS assessments. Rather, the main challenge facing such initiatives would appear to be competing pressures within a teacher’s workload, as over half of the sample stated that assessing FMS in schools would increase workload stress. This is perhaps unsurprising with research finding that teachers increasingly feel time pressured to cover the core curriculum (Routen et al., 2018). Using the behavior change techniques outlined by the behavior change wheel (Michie et al., 2011), future assessment tools should ensure that emotional support is available for school staff. This could be achieved by changing the culture in schools, by using a whole school approach to promoting FMS development and physical activity (Daly-Smith et al., 2020).

The results of this questionnaire have demonstrated that the physical and social constraints within a school aren’t compatible with the requirements of existing tests. Thus, as the school environment is unlikely to change, the nature of assessments must be adapted to suit (Michie et al., 2011). Current tools will therefore need to be revised, or new tools developed to account for the capacity issues that schools face and the constraints teachers perceive on their time. School-based FMS assessments should adhere to the following guidelines, which have been developed based on the results of this study: (i) assessments should be quick (30–60 min per class) and supported by high-quality face-to-face training which makes them straightforward to implement; (ii) a member of the SLT should be present and engaged with training to promote its value; (iii) manuals should be provided for schools which encourage an understanding of what FMS are and why they are important, as well as detailing how to implement the assessment; (iv) assessments should only utilize equipment that schools already have, or provide equipment for schools that will enable testing, (v) space constraints should be taken into account, ensuring that FMS can be assessed in a relatively small indoor or outdoor space (e.g., ≤5 m^2^); and (vi) teachers should be encouraged to set up a network of support within the school, to help ease workload stress, and encourage a healthy working environment. All of these factors will help align provisions available in schools, and help enable the assessment of FMS in schools to be sustainable (Daly-Smith et al., 2020).

It is important to recognize that this questionnaire, in common with all such surveys, could be subject to response bias. As the questionnaire was online, and optional, it is likely that participants who volunteered to take part had some interest in FMS and/or FMS assessments. Thus, the respondents of this study may have responded in a more optimistic manner than primary school teaching staff more generally. Research suggests that personality traits can influence use of social media sites (King et al., 2014; Rife et al., 2016) and that it can be difficult to validate participant identities (King et al., 2014) which may have influenced the generalizability of the results. However, it is widely acknowledged that the benefits of social media recruitment outweigh the limitations (King et al., 2014), and that online behavioral research can yield similar results to face-to-face equivalents (Casler et al., 2013). Moreover, due to the questionnaire being based online, it wasn’t possible to measure ‘actual’ physical capability, and thus the questionnaire needed to rely on perceived capability to assess whether teachers would have the necessary skills to demonstrate FMS and accurately measure pupils’ ability. These questions also did not provide a detailed explanation how each FMS would be performed, and thus, it is possible that there may have been some confusion over what was meant. For example, when asked about hopping, participants may have thought this referred to either hopping on one leg or bunny hopping. Additionally, the questionnaire did not ask about teachers’ understanding of how to interpret results of assessments, or how to help children who are identified as struggling with FMS development, two important factors which may influence motivation to assess FMS in schools. However, responses were mostly positive, with only circa 5% of teaching staff responding negatively to questions about the utility of FMS assessments for teachers, and likelihood to assess FMS in schools. Despite this, it will be important for any school-based assessment tool to ensure that teachers are equipped to understand and deal with the results they may obtain. Only one question was included which evaluated opportunity (social) as there is only one aspect of the TDF that links to this aspect of the COM-B model (social influences), future studies may wish to explore this aspect in more detail. In addition, the sample was relatively young – perhaps due to the manner in which the questionnaire was promoted, so the results may not accurately reflect the thoughts of older members of teaching staff. Finally, it is important to note that the validity and reliability of the questionnaire used in this study have not been tested. However, the authors ensured that questions were theoretically driven (i.e. aligned with FMS literature, the COM-B model and the TDF) and relevant for school teachers.

In conclusion, a large proportion of teaching staff in primary schools would assess FMS if they had the training but the majority lack the expertise to do so (primarily due to a lack of training). Equipment and opportunity do not appear to present barriers, with many predicting supportive senior leadership. It is likely that the lack of action relates to a lack of capacity to practically assess FMS in schools, due to time and training constraints of current assessments, together with the possibility of increased stress involved with needing to embed assessments alongside other provision. It is likely that current assessment tools are not acceptable, or feasible for use in schools, and thus more research is needed to modify existing measures, or develop new tools which take into account the key considerations (both acceptability and COM-B related) outlined in this paper.

## Data Availability

The data that support the findings of this study are available from the corresponding author, LHE, upon reasonable request.

## Publication statement

This manuscript has not been published elsewhere and not been submitted simultaneously for publication elsewhere.
